# Effect of Practicing Meditation, Pranayama, and Yoga on the Mental Health of Female Undergraduate Medical Students: An Interventional Study

**DOI:** 10.7759/cureus.28915

**Published:** 2022-09-07

**Authors:** Sunita ., Manju Lata, Himel Mondal, Manish Kumar, Raj Kapoor, Asha Gandhi

**Affiliations:** 1 Physiology, Indira Gandhi Institute of Medical Sciences, Patna, IND; 2 Physiology, Employees' State Insurance Corporation Medical College and Hospital, Patna, IND; 3 Physiology, Saheed Laxman Nayak Medical College, Koraput, IND; 4 Physiology, Vardhman Mahavir Medical College and Safdarjung Hospital, New Delhi, IND; 5 Physiology, Lady Hardinge Medical College and Associated Hospitals, New Delhi, IND

**Keywords:** female students, academic stress, psychological burnout, anger, anxiety, medical student, mental health, meditation, depression, yoga

## Abstract

Background

Medical education is a rigorous formal education with a vast syllabus. Burnout and stresses are common among medical students and more prominent in females. Female medical students suffer from a higher level of stress than male medical students. For the improvement of physical and mental health, meditation, pranayama, and yoga are some of the ancient techniques. Meditation is a technique of focusing the mind on a target like an object, activity, or any thought. Pranayama is an ancient yogic practice focusing on the breath. Yoga is a combination of physical, mental, and spiritual dimensions that has the potential to improve mental and physical health.

Objective

This study aimed to find the effect of meditation, pranayama, and yoga on the improvement of mental health among female undergraduate medical students.

Materials and methods

This was an interventional study. A total of 105 females with a median age of 19 years (first quartile - third quartile: 18-20) first-year undergraduate medical students were recruited for this study. They were randomly allocated to control, meditation, pranayama, and yoga groups. The control group did not practice any form of meditation, pranayama, or yoga. The rest of the group practiced a designated program for their group, six days a week for 12 weeks. The anxiety, depression, anger, and sense of well-being were assessed by a validated self-administered questionnaire developed by the Defence Institute of Physiology and Allied Sciences, New Delhi before starting the study, at six weeks, and at 12 weeks after the intervention. Inter-group levels of anxiety, depression, anger, and well-being were compared by the Kruskal-Wallis test with Dunn’s posthoc test. Intra-group parameters at baseline, at six weeks, and at 12 weeks after the intervention was tested by Friedman’s test.

Result

The age (years) (p = 0.07), height (cm) (p = 0.98), and weight (kg) (p = 0.26) of participants among groups were similar. Anxiety, depression, and anger significantly decreased after six weeks in all three intervention groups. A further decrement was seen after 12 weeks of meditation, pranayama, and yoga. The maximum effect was seen in the yoga group. A sense of well-being was improved after practicing all types of interventions. However, meditation was found to increase a sense of well-being to the highest level compared to pranayama and yoga.

Conclusion

Introduction and sustainment of meditation, pranayama, and yoga programs for first-year female undergraduate medical students may help reduce anxiety, depression, and anger and promote a sense of well-being. Although a six-week program helps to improve mental health, a 12-week program helps in further improvement. A yoga program is more effective for improving the mental health of the students in comparison with pranayama and meditation.

## Introduction

A physician must be physically and mentally healthy to lead a quality life to possess the virtue of altruism and empathy in patient care [1]. medical education is a rigorous and competitive professional course with a vast syllabus. Along with the stress of medical education during the long years of study, the care-demanding nature of the medical profession may be the cause of the neglect of own health [2]. The consequence of this neglect is evident in burnout, moral distress, compassionate fatigue, anxiety, sleep disorder, depression, and other spectra of mental disorders among medical students and doctors [3]. Previous studies have also revealed that female medical students suffer from a higher level of stress than male students. This may be attributed to the difference in coping strategies between males and females [4,5].

Yoga has a prominent position in the curriculum of the ancient Indian education system. The yoga techniques are ideal for preventive, promotive, curative, and rehabilitative health. Studies have shown that yogic practices help to cope with stress and create harmony within the self [6]. Yoga helps in tranquilizing the mind and rejuvenates self-esteem and confidence [7]. Psychological issues like anger, depression, anxiety, and other stress-related disorder like insomnia are associated with the lifestyle of medical professionals that can be reduced by yogic practices [8].

The precise practice of meditation, pranayama, and yoga requires time. However, undergraduate medical students, engaged in curricular and extracurricular activities may not invest adequate time in yogic practices. Hence, the current Indian medical undergraduate curriculum integrates yoga into the curriculum. The students get training and time for initiation of yoga [9]. However, the practice should be continued for a long time for the improvement of physical and mental health [7].

In this context, this study was conducted to find the effect of meditation, pranayama, and yoga program in reducing the levels of anxiety, depression, anger, and sense of well-being among undergraduate female medical students. Furthermore, the effect would be compared for a short-term (six weeks) and moderate-term (12 weeks) program for assessing the effect of duration on the improvement of mental health of undergraduate female medical students.

## Materials and methods

Ethics

This study involves human research participants. Potential participants were briefed about the study procedure, benefits, and risks. Then they were asked to participate voluntarily in this study. They were also informed that they can exit from the study at any moment without stating any reason for that. Then written informed consent was obtained from willing participants. This study was approved by the Institutional Ethics Committee of Lady Hardinge Medical College (reference number: LHMC/IEC/2004).

Study design and settings

This was an interventional study. The pre-intervention mental health was measured and then meditation, pranayama, and yoga programs were applied. After the completion of six weeks and 12 weeks, mental health was assessed with the same tool. This study was conducted in the Department of Physiology of Lady Hardinge Medical College, New Delhi, India.

Participants

**Figure 1:**
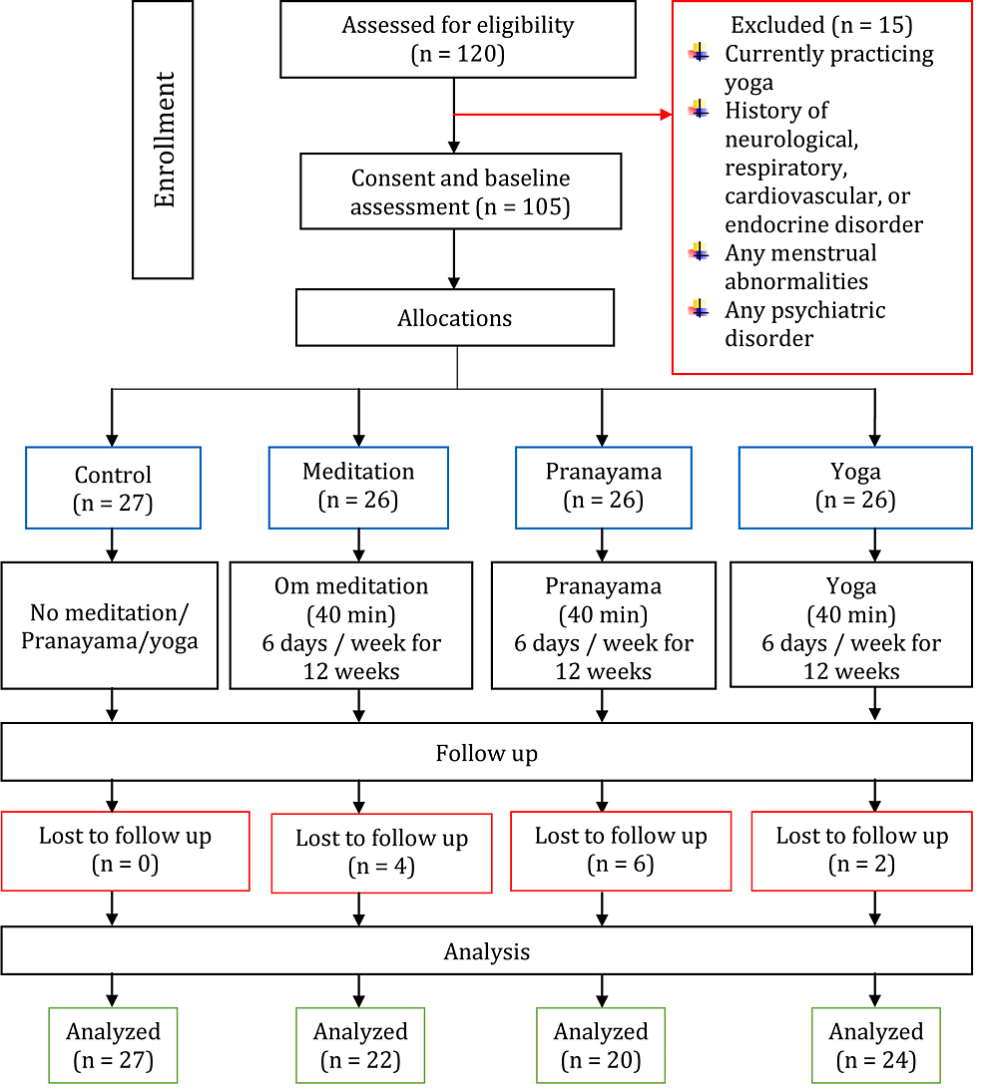
Research participants’ recruitment and their participation pattern in a flow chart

Female first-year medical students with a median age of 19 years (first quartile - third quartile: 18 - 20 years) studying pre-clinical subjects were recruited for this study. Any first-year female medical students providing written consent for voluntary participation were initially listed (n = 120). Then, the students who are currently or had previous exposure to meditation, pranayama, yoga, practicing any biofeedback (therapeutic measures to reduce stress by relaxation technique) or any other forms of relaxation technique, any present or history of respiratory ailments, cardiovascular, endocrine, and neuropsychiatric disorder or menstrual abnormalities like amenorrhea, oligomenorrhea, prolonged menstrual bleeding, dysmenorrhea were excluded from the study (n = 15). The enrolment and allocation are shown in Figure 1.

A total of 105 female participants were allocated equally to control, meditation, pranayama, and yoga groups randomly (block randomization for allowing an equal number of subjects in each group).

The intervention

The control group did not practice meditation, pranayama, or yoga in any form. The meditation group practiced Om meditation for 40 minutes, six days a week. The pranayama group practiced Nadi Shodhan Pranayama for 40 minutes, six days a week. The yoga group practiced a series of yoga events for 40 minutes, six days a week. All the participants continued the practiced it for a total of 12 weeks.

**Figure 2:**
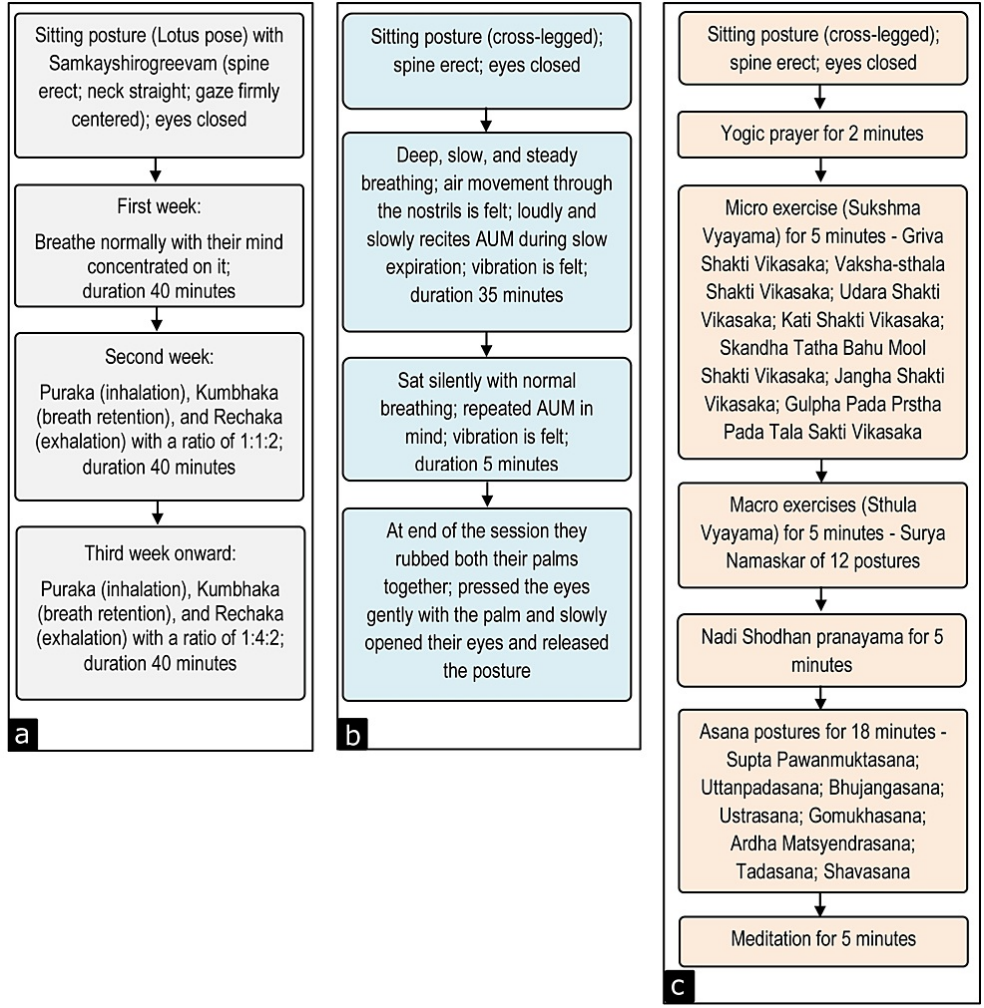
Brief procedure of programs Programs: (a) pranayama, (b) meditation, and (c) yoga All the programs were for 40 min each day, six days a week for 12 weeks

Nadi Shodhan Pranayama is a breathing exercise with purposeful control of breathing [10]. The Om meditation is a yogic meditation where the participants are relaxed and feel the vibration of “aum” chanting followed by a blissful silence [11]. Yoga is a combination of prayer, micro exercise, macro exercise, pranayama, asana, and meditation [12]. A flow chart of the brief procedure followed for pranayama (Figure 2a), meditation (Figure 2b), and yoga (Figure 2c) is shown in Figure 2.

The research participants were taught the techniques in the evening in a quiet room at a temperature of 25-28 °C. To ensure regularity and uniformity in practice, the training was provided by three trained meditation, pranayama, and yoga teacher (with an experience of > 5 years) throughout the study period. All the participants were called over the telephone one hour before the program. Those who were not reachable by telephone were sent a short message. Any participant who missed a total of > 3 sessions during a six-week program was excluded from the analysis.

Assessment of mental health

The Defence Institute of Physiology and Allied Sciences, New Delhi developed and validated a self-administered questionnaire to measure the level of anxiety, depression, anger, and sense of wellbeing. This questionnaire was used in a previous study involving Indian undergraduate medical students [13]. Hence, the questionnaire was deemed to be fit for our study participants. The questionnaire is available in English and Hindi and each question is typed in both languages side-by-side for a better understanding. The questionnaire has Likert-type response options where 0 indicates never and 3 indicate almost always. Anxiety is measured by 40 items, depression by 10 items, anger by 16 items, and sense of wellbeing by 50 items. The questionnaire is freely available for use for research purposes. This questionnaire was administered before starting the intervention, six weeks after the intervention, and 12 weeks after the intervention.

Statistical analysis

The data were first tested for distribution by the Shapiro-Wilk test. The majority of the data sets were found non-normally distributed. Hence, non-parametric statistical tests were used. Parameters before the intervention, at six weeks, and 12 weeks were tested by Friedman’s test. The inter-group comparison was conducted by the Kruskal-Wallis test. All tests were followed by a posthoc test to find the pair-wise difference. A p-value less than 0.05 was considered significant. We used GraphPad Prism 6.01 (GraphPad Software, USA) for conducting statistical tests.

## Results

The data of a total of 27 control, 22 meditation, 20 pranayama, and 24 yoga groups was analyzed (Figure 1). The age, height, weight, and BMI of the participants are shown in Table 1.

**Table 1:** Age, sex, and anthropometric parameters of participants *p-value of Kruskal-Wallis test

Variable	Control (n = 27)	Meditation (n = 22)	Pranayama (n = 20)	Yoga (n = 24)	p-value*
Age (years)	20 (19-20)	19 (18.75-20)	19 (19-20)	19 (19-20)	0.07
Height (cm)	158 (154-159.5)	157.5 (154.5-159.5)	156.25 (154.13-159.5)	156.5 (154.13-159.5)	0.98
Weight (kg)	61.9 (59.5-70.8)	59.5 (51.08-64.05)	59.5 (49.03-62.5)	60.6 (52.1-62.5)	0.26
BMI (kg/m^2^)	24.83 (23.27-26.69)	23.55 (20.05-26.66)	23.55 (19.98-25.66)	23.83 (20.55-26.51)	0.36

The anxiety score at baseline, after six weeks, and after 12 weeks of the program is shown in Table 2. There was a gradual decrement in the anxiety score over time after the initiation of the programs. At six weeks, there was no difference in anxiety scores among the three groups. However, at 12 weeks the score was lowest in the yoga group.

**Table 2:** Score of anxiety in control and three interventional groups before, after six weeks, and after 12 weeks of the program Scores expressed in median (first quartile - third quartile) * p-value of Friedman’s test (pair of data showing significant difference in posthoc test) † p-value of Kruskal-Wallis H test (pair of data showing significant difference in posthoc test) B: baseline. 6W: six-week program, 12W: 12-week program

Groups	Baseline	6 weeks	12 weeks	p-value*
Control	44 (38 - 46)	42 (39 - 44)	42 (36 - 49)	0.19
Pranayama	42 (38 - 45)	40 (36.25 – 43.75)	39 (36 – 43)	0.03 (B-12W)
Meditation	44.5 (38.75 – 47.25)	42 (39.75 – 45)	36.5 (32 – 40)	<0.0001 (B-12W, 6W-12W)
Yoga	39 (36.25 – 44)	39 (33 – 44.75)	31 (28 – 36)	<0.0001 (B-12W, 6W-12W)
p-value†	0.14	0.22	<0.0001 (C-M, C-I, M-I, P-I)	-

The depression score at baseline, after six weeks, and after 12 weeks of the program is shown in Table 3. There was a gradual decrement in the depression score over time after the initiation of the programs. At six weeks and 12 weeks, the depression score was lowest in the yoga group.

**Table 3:** Score of depression in control and three interventional groups before, after six weeks, and after 12 weeks of the program Scores expressed in in median (first quartile - third quartile) * p-value of Friedman’s test (pair of data showing significant difference in posthoc test) † p-value of Kruskal-Wallis H test (pair of data showing significant difference in posthoc test) B: baseline. 6W: six-week program, 12W: 12-week program

Groups	Baseline	6 weeks	12 weeks	p-value*
Control	7 (6 - 10)	8 (7 - 10)	8 (6 - 9)	0.12
Pranayama	7.5 (5 - 9)	6.5 (4 – 8)	5 (4 – 6)	<0.0001 (B-6W, B-12W, 6W-12W)
Meditation	8 (5 – 9)	7 (5 – 8)	4 (3 – 7)	<0.0001 (B-12W, 6W-12W)
Yoga	7 (4.25 – 8.75)	5.5 (4 – 7)	3 (2 – 5)	<0.0001 (B-6W, B-12W, 6W-12W)
p-value†	0.32	0.004 (C-I)	<0.0001 (C-P, C-M, C-I)	-

The anger score at baseline, after six weeks, and after 12 weeks of the program is shown in Table 4. There was a gradual decrement in the anger score over time after the initiation of the programs. At six weeks and 12 weeks, the depression score was lowest in the yoga group.

**Table 4:** Score of anger in control and three interventional groups before, after six weeks, and after 12 weeks of the program Scores expressed in median (first quartile - third quartile) * p-value of Friedman’s test (pair of data showing significant difference in posthoc test) † p-value of Kruskal-Wallis H test (pair of data showing significant difference in posthoc test) B: baseline. 6W: six-week program, 12W: 12-week program

Groups	Baseline	6 weeks	12 weeks	p-value*
Control	13 (11-17)	13 (10-18)	13 (10-19)	0.28
Pranayama	10.5 (9-13.75)	8.5 (7.25-13)	8 (6.25-9.75)	<0.0001 (B-12W, 6W-12W)
Meditation	14 (10.5-17.25)	10.5 (8.75-16)	8.5 (7.75-12.25)	<0.0001 (B-6W, B-12W, 6W-12W)
Yoga	10.5 (9-12)	9 (7.25-10)	6.5 (5-8)	<0.0001 (B-12W, 6W-12W)
p-value†	0.01 (C-I)	0.001 (C-P, C-I)	<0.0001 (C-P, C-M, C-I, M-I)	-

The sense of well-being score at baseline, after six weeks, and after 12 weeks of the program is shown in Table 5. There was gradual decrement (score decrement is an improvement of wellbeing) of the sense of wellbeing score over time after initiation of the programs. However, at the baseline, there was a difference among the groups and the meditation group had the lowest score. At six weeks and 12 weeks, the score was lowest in the meditation group.

**Table 5:** Score of sense of wellbeing in control and three interventional groups before, after six weeks, and after 12 weeks of the program Scores expressed in median (first quartile - third quartile) * p-value of Friedman’s test (pair of data showing significant difference in posthoc test) † p-value of Kruskal-Wallis H test (pair of data showing significant difference in posthoc test) B: baseline. 6W: six-week program, 12W: 12-week program

Groups	Baseline	6 weeks	12 weeks	p-value*
Control	44 (41-51)	45 (37-51)	45 (38-51)	0.65
Pranayama	51 (42.5 - 58.25)	48.5 (41-56.75)	43.5 (38.5-52.75)	<0.0001 (B-12W, 6W-12W)
Meditation	46.5 (41.75 - 52.25)	41 (36-45.5)	30 (23-39)	<0.0001 (B-6W, B-12W, 6W-12W)
Yoga	56 (45-61)	52 (44.5-57.75)	40 (36.25-42.75)	<0.0001 (B-12W, 6W-12W)
p-value†	0.01 (C-I, M-I)	0.002 (P-M, M-I)	<0.0001 (C-M, P-M, M-I)	-

## Discussion

To find the effect of meditation, pranayama, and yoga program on the mental health of female medical students, we found that the practice of any one among the three can reduce anxiety, depression, and anger, and promote a sense of well-being. However, among the three forms, yoga has a more prominent effect than the other two. The current study supports the fact that yoga has the potential to improve physical, mental, and spiritual health. However, in the current study, we evaluated the effect of the programs on mental health and did not observe the effect on physical or spiritual health.

One of the key objectives of yoga is to establish mental tranquillity to foster well-being, relaxation, less irritation, and an upbeat attitude toward life [14]. When a person practices yoga, the posterior hypothalamus is inhibited which improves the body's sympathetic reactions to stressful stimuli. It also helps to recover the stress-related autonomic regulating reflex systems. In addition, the reward centers in the central nervous system are stimulated and that causes suppression of the brain regions responsible for fear, aggression, and fury [15]. Hence, yoga can alleviate stress and lead to a feeling of happiness and pleasure. Regular yoga practice not only improves mental health, but can also help to reduce the heart rate, respiratory rate, and blood pressure [15].

Regular physical activity and mental relaxation techniques are one of the most important non-pharmacological ways of controlling cardiovascular and mental health. Studies have reported that yoga can improve depression in patients suffering from bipolar disorder [16]. It also helps in reducing attention-deficit hyperactivity disorder [17]. Patients, where exercise is helpful for the improvement of their condition, can enroll in a yoga program that would help them to continue the physical exercise in a specialized group that would also provide a sense of inclusion [18]. Yoga is not only a therapeutic tool but can be a preventive tool to combat mental and physical health challenges. In the current study, we included undergraduate medical students who were apparently healthy (physically). After practicing meditation, pranayam, or yoga, they had less anxiety, depression, and anger. Hence, these yogic programs are not only helpful for patients but can be helpful for otherwise normal individuals.

A study by Lemay et al. found that a 6-week 60-minute yoga program with meditation can reduce stress and anxiety levels among college students [19]. Yadav et al. reported that a short-term yoga program helps reduce stress and promote a sense of well-being among Indian undergraduate medical students [20]. Supporting these studies, we additionally found that although a short-term (six weeks in our study) program is helpful in improving mental health, a sustained yoga program for more time further improves mental health. Hence, the mediation, pranayam, or yoga program may be continued for maintaining the mental health of the medical students. Stakeholders may think about incorporating a guided program into the undergraduate medical curriculum.

Limitation of the study

This was a small-scale study conducted in a single medical institute situated in the northern zone of India. The pattern of psychological distress may differ according to geographical position and institutional organization and management. The research participants were recruited from first-year medical students. Their level of stress would be higher than in the other years [21]. Hence, similar programs in students in other years may show deviation in finding. We used a self-administered questionnaire that only records the perceived status of mental health. We did not use any biological marker of stress due to logistics limitations. These limitations should be considered by other researchers while conducting a similar study in their settings.

## Conclusions

Meditation, pranayama, or yoga programs for female undergraduate medical students may help reduce their levels of anxiety, depression, and anger. It also helps to promote a sense of well-being. Yoga has the most potential to enhance mental wellness when compared to the other two forms. Any of the three types of yogic practice for a period of six weeks may improve the mental health of female medical students. However, a longer period of yoga practice (12 weeks) may help further improve mental health. This finding would help implement a suitable program for undergraduate medical students by the stakeholders for helping students cope with anxiety, depression, and anger in a new academic environment.
